# Safety evaluation of the long-term intake of heat-killed *Lactobacillus helveticus* MCC1848 in healthy adults: a randomized, double-blind, placebo-controlled study

**DOI:** 10.1186/s40795-026-01379-0

**Published:** 2026-06-02

**Authors:** Takahiro Aoki, Natsumi Mutoh, Satoshi Arai, Noriyuki Iwabuchi, Yoshitaka Iwama, Miyuki Tanaka

**Affiliations:** 1 Biotics Research Institute, R&D Division, Morinaga Milk Industry Co., Ltd, 5-1-83, Higashihara, Zama 252-8583 Japan; 2 Nihonbashi Cardiology Clinic, Anera Bldg. 8F, 3-3-3 Higashi Nihonbashi, Chuo-ku, Tokyo, 103-0004 Japan; 3 Food Function Research Institute, R&D Division, Morinaga Milk Industry Co., Ltd, 5-1-83, Higashihara, Zama 252-8583 Japan

**Keywords:** *Lactobacillus helveticus*, Postbiotics, Safety, Clinical trial

## Abstract

**Background:**

Heat-killed *Lactobacillus helveticus* MCC1848 is a postbiotic, which potentially aids in maintaining a positive mood. Daily intake of heat-killed *L. helveticus* MCC1848 for 4 weeks by healthy adults is known to be safe. However, the safety of long-term *L. helveticus* MCC1848 intake has not been investigated.

**Methods:**

This study aimed to evaluate the safety of long-term intake of 25 billion heat-killed *L. helveticus* MCC1848 in healthy adults through a randomized controlled trial involving 40 participants. The study participants were randomly assigned to either the MCC1848 group (*n* = 20) or placebo group (*n* = 20). During a 12-week period, the MCC1848 group received the test powder containing 25 billion cells of heat-killed *L. helveticus* MCC1848, which is five times the minimum effective dose suggested for maintaining a positive mood. The placebo group received dextrin powder without any detectable heat-killed *L. helveticus* MCC1848. Safety evaluations included anthropometric and blood pressure measurements, hematological and biochemical tests, urinalysis, and adverse event monitoring.

**Results:**

No clinical issues were observed in either of the biochemical evaluations. Furthermore, no adverse events were reported due to the intake of heat-killed *L. helveticus* MCC1848.

**Conclusions:**

The results of this study suggest that 25 billion heat-killed *L. helveticus* MCC1848 for 12 weeks is safe for intake in healthy adults.

**Trial Registration:**

The clinical trial was registered as UMIN000055233 (Registration Date: August 13, 2024) in the Clinical Trials Registry of the University Hospital Medical Information Network.

## Background

In contemporary society, the prevalence of individuals experiencing stress in their daily lives is increasing [1]. Chronic stress is widely recognized to worsen mood states and contribute to a decline in quality of life (QOL) [2]. To mitigate these issues, the development of novel intervention strategies aimed at managing stress and enhancing QOL is imperative.

Recently, there has been growing interest in the gut-brain axis, with studies suggesting that probiotics and their heat-killed forms (postbiotics) may ameliorate mood states such as stress and anxiety [3–5]. Research in this domain is attracting considerable attention alongside the expansion of the anti-stress market and is anticipated to gain increasing significance [6]. For such products to be used widely as functional foods or dietary supplements, however, not only evidence of efficacy but also a clear understanding of their safety and tolerability is essential, particularly when they are considered in GRAS‑related regulatory contexts.

*L. helveticus* MCC1848 (strain happiness) is a strain of lactic acid bacteria that has been reported to exhibit various health-promoting functions in its heat-killed form [7–9]. Due to its heat-killed state, it is highly safe and can be seamlessly incorporated into various food products. This characteristic has garnered attention in the food and health food industries. Moreover, *L. helveticus*, the species to which MCC1848 belongs, is included in the European Food Safety Authority’s Qualified Presumption of Safety (QPS) list [10], supporting its suitability for use in food applications. Although the QPS concept primarily pertains to viable microorganisms, using a heat‑killed preparation further reduces safety concerns associated with bacterial proliferation while maintaining functional components, and thus is particularly advantageous from both technological and regulatory perspectives.

Animal studies have demonstrated anxiolytic effects of heat-killed *L. helveticus* MCC1848 [7], and preliminary human trials have suggested QOL improvement effects such as anti-stress and anti-fatigue [8]. Furthermore, randomized controlled trials have indicated that consuming 5 billion heat-killed *L. helveticus* MCC1848 cells daily for four weeks may help sustain a positive mood state [9]. While these findings support the functional potential of heat‑killed *L. helveticus* MCC1848, they are based on short‑term intake at relatively modest doses, and there remains a lack of data on the effects of long‑term, high‑dose consumption in humans.

Because heat‑killed *L. helveticus* MCC1848 can be readily formulated into a wide range of food products and supplements, it is plausible that consumers may ingest amounts exceeding the previously reported effective dose in real‑world settings. Therefore, assessing the safety and tolerability of long‑term, high‑dose intake under conditions that reflect potential consumer exposure is an important prerequisite for its broader use as a postbiotic ingredient. In the present study, we selected a daily dose of 25 billion cells, which corresponds to five times the minimum effective dose (5 billion cells/day) reported by Mutoh et al. to sustain positive mood states [8]. This five‑fold dose is also consistent with Japanese guidelines for the safety evaluation of foods with function claims, which recommend conducting human safety studies at 3–5 times the effective dose [11]. Accordingly, the chosen dose is supported by both scientific evidence and regulatory guidance.

To address the current evidence gap, this study aims to evaluate the impact of long-term high-dose intake of *L. helveticus* MCC1848 in humans. The specific objective was to ascertain the safety and tolerability of consuming 25 billion heat‑killed *L. helveticus* MCC1848 cells daily for 12 weeks in healthy adults.

## Methods

### Study design and settings

This randomized, double-blind, placebo-controlled, parallel-group clinical trial was conducted at the Nihonbashi Cardiology Clinic in Tokyo from September 2024 to January 2025. The present study followed the CONSORT (Consolidated Standards of Reporting Trials) guidelines. The study received approval from the Ethics Committee of the Kobuna Orthopedic Clinic (Approval No.: MK-2408-01, Approval Date: August 13, 2024). The study design adhered to the principles of the Declaration of Helsinki and the “Ethical Guidelines for Medical and Health Research Involving Human As” (Ministry of Education, Culture, Sports, Science and Technology, Ministry of Health, Labor, and Welfare). Prior to the study, written informed consent was obtained from all the participants following a comprehensive explanation of the purpose and procedures. The trial was registered as UMIN000055233 (Registration Date: August 13, 2024, URL: https://center6.umin.ac.jp/cgi-open-bin/ctr/ctr.cgi?function=brows&action=brows&recptno=R000063103&type=summary&language=J) in the Clinical Trials Registry of the University Hospital Medical Information Network prior to participant enrollment. In addition, there were no revisions to the protocol after study initiation. The study protocol and statistical analysis plan were finalized before recruitment began, and no modifications were made after the trial commenced. The trial was conducted in full accordance with the predefined procedures, and no protocol deviations occurred. The primary objective of this trial was to evaluate safety and tolerability. Accordingly, all outcome measures—anthropometric and blood pressure measurements, hematological and biochemical parameters, urinalysis, and adverse event monitoring—were prespecified as safety endpoints.

### Study participants

The participants were enrolled according to stringent inclusion and exclusion criteria. The inclusion criteria targeted healthy adult volunteers aged 18 to 64 years. Following were the exclusion criteria: (1) Individuals with severe complications or diseases requiring urgent treatment; (2) Individuals with chronic diseases under regular medication; (3) Individuals who could not discontinue foods or supplements containing lactic acid bacteria, bifidobacteria, oligosaccharides, etc., during the study period; (4) Individuals with gastrointestinal diseases which affect digestion and absorption, or those with a history of gastrointestinal surgery (excluding appendicitis); (5) Individuals with a history or current condition of dependence on drugs or alcohol; (6) Individuals currently participating or intending to participate in other food or drug intake trials, or trials involving the application of cosmetics or pharmaceuticals; (7) Individuals with a history of drug allergies or severe food allergies; (8) Pregnant individuals or those who intend to become pregnant during the study period or those breastfeeding; (9) Heavy alcohol consumers (average daily pure alcohol intake equal to 20 g or more); (10) Heavy smokers (21 or more cigarettes per day); 11. Individuals who donated 400 mL or more blood within 12 weeks before the start of intake, or those who donated 200 mL or more of blood during the pre-observation period; 12. Individuals who tested positive for infectious diseases during screening tests; 13. Individuals deemed inappropriate by the principal investigator or sub-investigator based on background, physical findings, examination, blood pressure, pulse rate, and clinical test results.

Participants were randomly assigned to receive heat-killed *L. helveticus* MCC1848 (MCC1848 group) or placebo in a 1:1 ratio using a computer-generated random number list with a randomly permuted block method. The randomization list and allocation table were prepared and managed by an independent staff member who was not involved in the conduct of the trial. Both investigators and participants remained blinded to group assignments until the database was locked. To maintain blinding integrity, the test food and placebo were indistinguishable in packaging, appearance, taste, and aroma, ensuring that both participants and outcome assessors were fully blinded throughout the study. The target number of cases was set at 20 participants per group to aid statistical evaluation.

### Study products

Participants received a placebo (maltodextrin powder) or *L. helveticus* MCC1848 powder, which was packed into sachets containing 2.5 × 10^10^ heat-killed cells, which is five times the amount used by Mutoh, et al. [8], with maltodextrin as the excipient. The participants consumed one sachet pack daily. The test powder used in this study was manufactured by Morinaga Milk Industry Co., Ltd. (Tokyo, Japan). The test product is equivalent to the commercially available heat-treated MCC1848 powder produced by the company. The test foods were stored at room temperature under dry, light‑protected conditions throughout the trial. Pre‑trial stability testing confirmed that the cell count and physicochemical properties of the heat‑killed MCC1848 powder remained stable over the duration corresponding to the study period, ensuring maintenance of product integrity.

### Measurements

The study included a two-week pre-intake observation period, a 12-week intervention period, and a four-week post-intake observation period. Safety and tolerability were assessed comprehensively through anthropometric and blood pressure measurements, blood hematology and biochemistry, urinalysis, and systematic recording of adverse events and side effects. During the intervention period, the participants ingested the heat-killed *L. helveticus* MCC1848 powder or placebo powder daily with water or lukewarm water. They were instructed to avoid significant dietary or lifestyle changes, excessive alcohol consumption, and overeating during the study period. Participants recorded daily intake of the study product, physical condition, medication use, and medical visits in a study diary, which was reviewed at each visit to monitor adherence. In addition, individuals who could not discontinue foods or supplements containing lactic acid bacteria, bifidobacteria, or oligosaccharides during the intervention were excluded at screening to prevent concomitant intake of other probiotics or prebiotics. The participants visited the clinic for screening 5 weeks before experiment initiation at week 0 at the start of the intervention, at weeks 4, 8, and 12 during the intervention, and at week 16 after the follow-up period. During these visits, the participants underwent interviews, anthropometric measurements, blood pressure measurements, and provided blood and urine samples. Unfavorable or unintended events which occurred during the study period were defined as adverse events (AEs). Specifically, the principal investigator evaluated the events based on the participant’s condition during the visit, the details in the participant’s diary on changes in physical condition, hospital visits, treatment, and the results of blood and urine tests, as well as the situation before intake and the range of variation for each reference value. The principal investigator conducted interviews to assess the participants’ physical condition, subjective symptoms, and AEs during the study period.

Height was measured only during screening. Body weight (BW), systolic blood pressure (SBP), diastolic blood pressure (DBP), and pulse rate were measured at all visits. BW was measured using a scale (HD-662, TANITA, Tokyo, Japan) capable of measuring up to 150 kg. During the BW measurement, participants wore light clothing and no footwear. Body mass index (BMI) was calculated as body weight (kg) divided by height (m^2^). Blood pressure and pulse rate were measured in a seated position using an automated blood pressure monitor (HEM-907, Omron Corporation, Kyoto, Japan).

Blood and urine analyses were performed at all visits. Hematological tests recorded white blood cell count, red blood cell count (RBC), hemoglobin (Hb), hematocrit, platelet count, mean corpuscular volume (MCV), mean corpuscular hemoglobin (MCH), mean corpuscular hemoglobin concentration (MCHC), and white blood cell differentiation (percentages of neutrophils, lymphocytes, monocytes, eosinophils, and basophils). Biochemical tests recorded total protein, albumin (ALB), total bilirubin (TB), aspartate transaminase (AST), alanine aminotransferase (ALT), lactate dehydrogenase (LD; IFCC), alkaline phosphatase (ALP; IFCC), γ-glutamyl transferase, blood urea nitrogen (BUN), creatinine (CRE), uric acid, sodium (Na), chloride (Cl), potassium (K), calcium (Ca), total cholesterol (TC), LDL-cholesterol (LDL-C), HDL-cholesterol (HDL-C), triglycerides, fasting blood glucose (FBG) and hemoglobin A1c (HbA1c). Urinalysis measured urine specific gravity (USG), urine pH (U-pH), urine protein (U-pro), urine glucose (U-glu), urine urobilinogen (U-uro), urine bilirubin (U-bil), urine ketones (U-ket), and occult blood reaction (OBR). LSI Medience Corporation (Japan) performed the hematological tests, blood biochemical tests, and urinalysis.

All participants were monitored for AEs during the study period. Safety monitoring included questionnaires on general health status and the occurrence of health-related events. The principal investigator reviewed the interviews at weeks 0, 4, 8, 12, and 16, along with the data from the participants’ diaries. The principal investigator conducted case reviews while maintaining blinding of group allocation and determined the relationship between any AE and intake of the study products. Causality was assessed with reference to the Common Adverse Events Reporting Guideline in Japanese Cancer Trial Network (JCTN), which categorizes adverse events as “Definite,” “Probable,” “Possible,” “Unlikely,” or “Not related.”

### Statistical analysis

Statistical analyses were conducted based on the full data set, defined as all randomized participants who received study treatment and had at least one post-treatment test result. Those who dropped out were treated as missing. Values are presented as mean ± standard deviation. Comparisons between the MCC1848 and placebo group at each test time point were statistically analyzed using an unpaired t-test. Comparisons of test results within groups were analyzed using a paired t-test. For categorical data, statistically significant differences between the study groups were examined using Fisher’s exact test. For urinalysis data, except for specific gravity and pH, the data were coded as 0 or 1, representing within or outside the reference range, respectively, and presented as a matrix of participant numbers and codes, which were analyzed using Fisher’s exact test. AEs were tabulated for each group. The Fisher exact test was performed for the incidence rate and *p* value was calculated. The results were considered significant at *p* < 0.05 according to the two-sided tests. All statistical analyses were performed using IBM SPSS Statistics version 27 (IBM Corp.).

## Results

### Study design

**Fig. 1:**
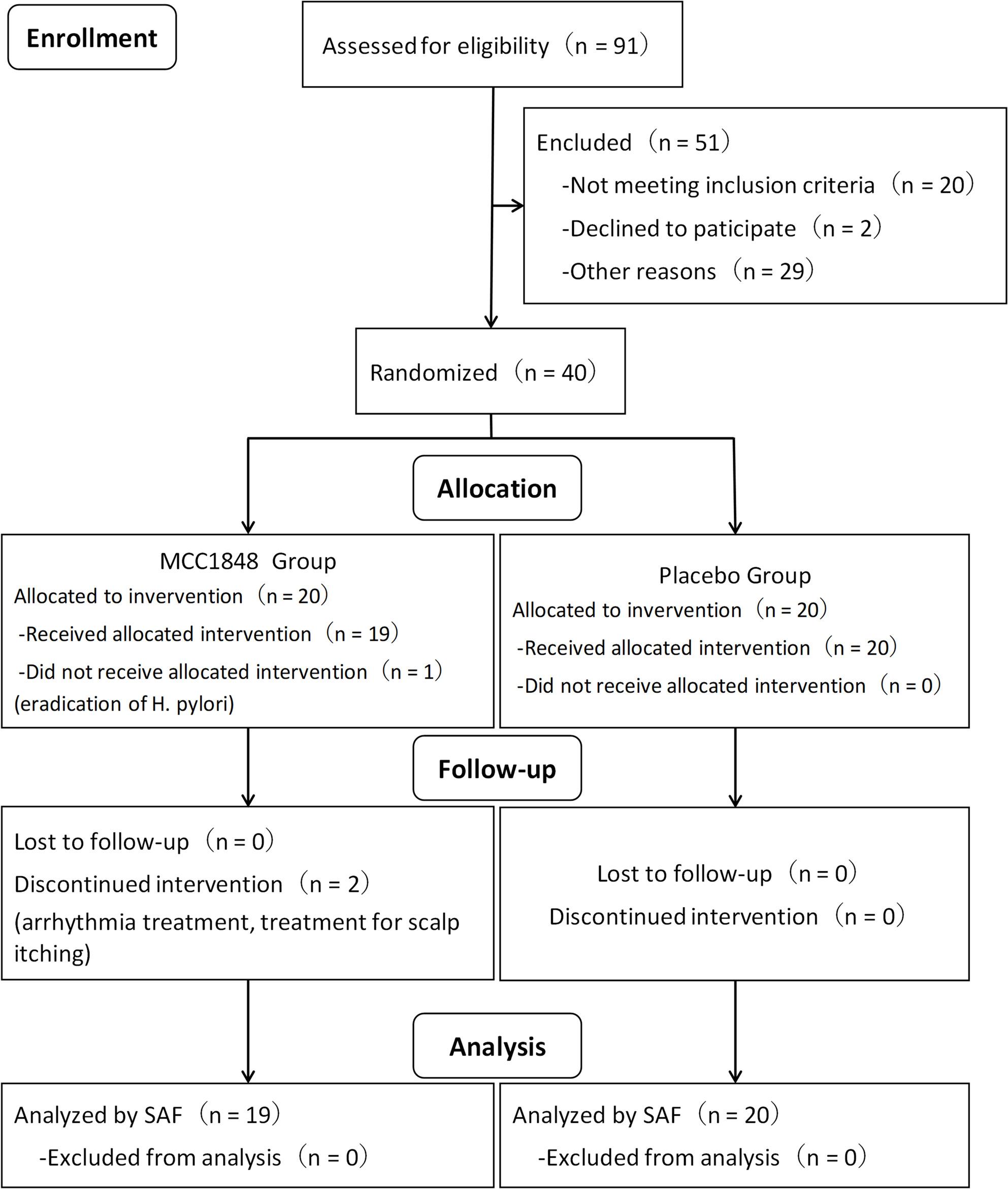
Study flow diagram

Of a total of 91 participants screened for this trial, 40 participants (47.6 ± 9.6 years old, 14 males and 26 females) were enrolled and randomly assigned to either the MCC1848 group (*n* = 20) or the placebo group (*n* = 20) (Fig. 1). One participant was excluded before trial initiation due to the eradication of *H. pylori*, one participant was excluded by week 4 due to arrhythmia treatment, and one participant was excluded at week 8 due to treatment for scalp itching due to hair dressing use; all the other participants completed the trial. The safety analysis was conducted on participants who had consumed the test foods at least once, in the Safety Analysis Set (SAF) population consisting of 20 participants in the placebo group and 19 participants in the MCC1848 group (47.1 ± 10.4 years old, 13 males, 26 females). The characteristics of the participants at SAF are shown in Table 1.

**Table 1:** Participant characteristics in the safety analysis set

Characteristics	MCC1848 (*n* = 19)	Placebo (*n* = 20)
Male/Female (*n*)	6/13	7/13
Age (years) [range]	47.1 ± 10.4 [23–60]	47.8± 9.3 [28–62]
Height (cm) [range]	162.7 ± 7.6 [149.0–180.0]	165.6 ± 8.1 [151.9–179.6]
BW (kg) [range]	58.5 ± 6.8 [48.3–73.4]	60.0 ± 8.7 [48.0–80.5]
BMI (kg/m^2^) [range]	22.1± 1.9 [18.6–25.3]	21.8± 2.2 [19.1–25.9]
SBP (mmHg) [range]	121.8 ± 12.5 [102–146]	122.2 ± 13.8 [101–148]
DBP (mmHg) [range]	78.4 ± 10.5 [65–101]	74.7 ± 14.4 [53–104]
Pulse rate (bpm) [range]	74.6 ± 9.6 [54–95]	72.8 ± 10.0 [52–89]

Data represent number of subjects or means ± standard deviations

*Abbreviations*: *BW *Body weight, *BMI *Body mass index, *SBP *Systolic blood pressure, *DBP *Diastolic blood pressure

### Anthropometric and blood pressure measurements

Table 2 summarizes the anthropometric and blood pressure measurements at baseline (week 0), during treatment (weeks 4, 8, and 12), and after treatment (week 16). The following results present the mean differences with 95% confidence interval (CI). At week 0, DBP was significantly higher in the MCC1848 group than that in the placebo group (7.0 [1.44, 12.56] mmHg, *p* = 0.015). Apart from this difference in diastolic blood pressure, no notable baseline imbalances were observed between the two groups in any other anthropometric or clinical parameters. In the MCC1848 group, the pulse rate was significantly lower at weeks 12 and 16 compared to that at week 0 (-4.65 [-8.08, -1.21], -6.18 [-9.59, -2.76] beats/min, *p* = 0.011, *p* = 0.001, respectively). BW and BMI at week 12 were significantly higher than those at week 0 (0.72 [0.07, 1.37] kg, 0.27 [0.04, 0.50] kg/m^2^, *p* = 0.031, *p* = 0.025, respectively), and DBP at week 16 was significantly higher than that at week 0 (3.71 [0.58, 6.83] mmHg, *p* = 0.023). In the placebo group, the BW at week 12 was significantly higher than that at week 0 (0.55 [0.03, 1.07] kg, *p* = 0.040). SBP in the placebo group was significantly higher at week 16 compared to that at week 0 (5.80 [0.31, 11.3] mmHg, *p* = 0.040), and DBP was significantly higher at weeks 4 and 16 compared to that at week 0 (3.00 [0.27, 5.73], 7.60 [3.20, 12.0] mmHg, *p* = 0.033, *p* = 0.002, respectively).

**Table 2:** Summary of anthropometric and blood pressure measurements

Variables	Group	Week 0	Week 4	Week 8	Week 12	Week 16	
n(number)	MCC1848	19	18	17	17	17	
	Placebo	20	20	20	20	20	
BW (kg)	MCC1848	58.8 ± 6.9	59.5 ± 6.8	59.9 ± 7.1	60.1 ± 7.1*	60.1 ± 7.1	
	Placebo	60.4 ± 9.2	60.4 ± 9.3	60.3 ± 9.6	61.0 ± 9.6*	60.9 ± 9.5	
BMI (kg/m^2^)	MCC1848	22.2 ± 1.9	22.4 ± 1.7	22.7 ± 1.8	22.8 ± 1.7*	22.8 ± 1.7	
	Placebo	21.9 ± 2.3	21.9 ± 2.3	21.9 ± 2.5	22.1 ± 2.5	22.1 ± 2.4	
SBP (mmHg)	MCC1848	119.3 ± 11.5	117.5 ± 13.0	116.3 ± 14.0	124.4 ± 14.4	124.3 ± 14.0	
	Placebo	115.5 ± 12.8	115.1 ± 14.1	113.1 ± 13.1	119.3 ± 17.0	121.3 ± 17.2*	
DBP (mmHg)	MCC1848	73.9 ± 6.8†	74.2 ± 8.8	73.9 ± 9.9	76.7 ± 10.6	77.9 ± 10.5*	
	Placebo	67.0 ± 10.0	70.0 ± 12.7*	68.7 ± 12.0	70.6 ± 14.4	74.6 ± 15.5**	
Pulse rate (bpm)	MCC1848	77.8 ± 8.3	78.6 ± 9.1	76.2 ± 8.2	73.9 ± 9.8*	72.4 ± 6.8**	
	Placebo	78.2 ± 8.0	76.0 ± 11.0	76.6 ± 11.5	77.9 ± 11.5	76.7 ± 10.0	

Data represent means ± standard deviations

*Abbreviations*: *BW *Body weight, *BMI *Body mass index, *SBP *Systolic blood pressure, *DBP *Diastolic blood pressure

* *p* < 0.05 and ** *p* < 0.01 indicate significant difference compared with week 0 values using t-test; † *p* < 0.05 indicates significant difference compared with the placebo group using unpaired t-test

### Blood and urine analysis

Tables 3 and 4 summarize the results of the hematological tests. The following results present the mean differences with 95%CI. In the MCC1848 group, Ht levels at week 4 were significantly lower than those at week 0 (− 1.39 [− 2.04, − 0.74] %, *p* = 0.0003), and MCV at weeks 4, 8, 12, and 16 was significantly lower than that at week 0 (− 2.06 [− 2.90, − 1.21], − 1.29 [− 2.44, − 0.15], − 1.59 [− 2.68, − 0.50], − 2.24 [− 3.29, − 1.18] fl., *p* = 0.0001, *p* = 0.003, *p* = 0.007, *p* = 0.0004, respectively). Hb levels at week 12 were significantly higher than those at week 0 (0.35 [0.04, 0.67] g/dL, *p* = 0.003); MCH levels at week 4 and 8 were significantly higher than those at week 0 (0.60 [0.25, 0.95], 0.24[0.003, 0.48] pg, *p* = 0.002, *p* = 0.047, respectively); MCHC levels at weeks 4, 8, 12, and 16 were significantly higher compared to those at week 0 (1.29 [0.92, 1.67], 0.68 [0.37, 0.98], 0.62 [0.23, 1.01], 0.91 [0.57, 1.24] %, *p* = 0.000001, *p* = 0.0002, *p* = 0.004, *p* = 0.00003, respectively).

**Table 3:** Summary for the findings of hematological examination (blood cell count)

Variables	Reference range	Group	Week 0	Week 4	Week 8	Week 12	Week 16
n(number)		MCC1848	19	18	17	17	17
		Placebo	20	20	20	20	20
WBC (×10^2^/µl)	33–90	MCC1848	51.1 ± 13.3	51.8 ± 15.2	50.9 ± 16.8	51.9 ± 13.1	50.0 ± 15.7
		Placebo	57.0 ± 13.0	56.2 ± 13.7	57.2 ± 15.3	55.0 ± 13.0	56.5 ± 15.6
RBC (×10^4^/µl)	M: 430–570	MCC1848	456.4 ± 36.2	450.3 ± 35.8	457.3 ± 35.3	466.1 ± 37.2	456.7 ± 26.4
	F: 380–500	Placebo	455.3 ± 38.6	449.6 ± 42.4	462.3 ± 37.5	468.3 ± 41.0**	459.6 ± 46.0
Hb (g/dl)	M: 13.5–17.5	MCC1848	13.7 ± 0.8	13.8 ± 0.8	13.8 ± 0.8	14.0 ± 1.0*	13.8 ± 0.8
	F: 11.5–15.0	Placebo	13.3 ± 1.5	13.3 ± 1.6	13.6 ± 1.4*	13.6 ± 1.6*	13.4 ± 1.7
Ht (%)	M: 39.7–52.4	MCC1848	43.7 ± 2.6	42.3 ± 2.8**	43.2 ± 2.6	44.0 ± 3.3	42.7 ± 2.2
	F: 34.8–45.0	Placebo	42.4 ± 3.7	41.5 ± 4.2*	42.5 ± 3.6	43.0 ± 3.8	42.1 ± 4.3
PLT (×10^4^/µl)	14.0–34.0	MCC1848	25.0 ± 5.1	25.1 ± 5.8	25.5 ± 6.1	26.1 ± 5.8	26.4 ± 8.1
		Placebo	26.3 ± 6.5	26.5 ± 6.0	26.3 ± 7.5	27.2 ± 6.6	26.9 ± 7.5
MCV (fl.)	85–102	MCC1848	96.1 ± 4.1	94.1 ± 3.4**	94.6 ± 3.0*	94.4 ± 3.2**	93.7 ± 3.3**
		Placebo	93.2 ± 5.0	92.4 ± 5.0*	91.9 ± 5.0**	92.0 ± 5.2**	91.6 ± 4.7**
MCH (pg)	28.0–34.0	MCC1848	30.0 ± 1.4	30.6 ± 1.4**	30.2 ± 1.4*	30.1 ± 1.4	30.2 ± 1.5
		Placebo	29.3 ± 2.3	29.7 ± 2.4**	29.3 ± 2.3	29.1 ± 2.4	29.2 ± 2.2
MCHC (%)	30.2–35.1	MCC1848	31.2 ± 0.8	32.6 ± 1.1**	32.0 ± 0.7**	31.9 ± 0.8**	32.2 ± 0.8**
		Placebo	31.4 ± 1.2	32.1 ± 1.3**	31.9 ± 1.0**	31.6 ± 1.2	31.9 ± 1.3**

Data represent means ± standard deviations

*Abbreviations*: *M *Male, *F *Female, *WBC *White blood cell count, *RBC *Red blood cell count, *Hb *Hemoglobin, *Ht *Hematocrit, *PLT *Platelet count, *MCV *Mean corpuscular volume, *MCH *Mean corpuscular hemoglobin, *MCHC *Mean corpuscular hemoglobin concentration

* *p* < 0.05 and ** *p* < 0.01 indicate significant difference compared with week 0 values using paired t-test

**Table 4:** Summary of hematological examination (differential leukocyte ratio)

Variables	Reference range	Group	Week 0	Week 4	Week 8	Week 12	Week 16
n(number)		MCC1848	19	18	17	17	17
		Placebo	20	20	20	20	20
Neut (%)	40.0–75.0	MCC1848	55.3 ± 7.8	56.6 ± 6.8	57.2 ± 7.8	57.8 ± 8.0	54.9 ± 8.2
		Placebo	54.8 ± 7.9	56.6 ± 8.8	58.0 ± 9.3	57.9 ± 8.5*	54.3 ± 9.4
LYMPH(%)	18.0–49.0	MCC1848	34.7 ± 7.7	33.8 ± 6.1	33.4 ± 7.2	32.7 ± 6.4	35.4 ± 7.1
		Placebo	35.3 ± 7.6	33.6 ± 8.4	32.4 ± 8.5*	32.3 ± 8.0*	35.7 ± 8.5
Mono (%)	2.0–10.0	MCC1848	6.0 ± 1.4	5.4 ± 1.4	5.4 ± 1.0	5.4 ± 1.5	5.8 ± 1.2
		Placebo	5.8 ± 0.9	5.9 ± 1.3	5.8 ± 1.2	5.6 ± 1.2	6.4 ± 1.3
Eosino (%)	0.0–8.0	MCC1848	3.2 ± 1.9	3.4 ± 2.6	3.1 ± 2.6	3.2 ± 2.4	3.0 ± 2.1
		Placebo	3.1 ± 1.8	3.1 ± 1.7	2.9 ± 1.6	3.2 ± 1.7	2.7 ± 1.0
Baso (%)	0.0–2.0	MCC1848	0.9 ± 0.3	0.8 ± 0.5	0.9 ± 0.5	0.9 ± 0.4	0.9 ± 0.4
		Placebo	0.9 ± 0.4	0.9 ± 0.4	0.9 ± 0.4	0.9 ± 0.4	0.9 ± 0.3

Data represent means ± standard deviations

*Abbreviations*: *Neut *Neutrophils/leukocytes, *LYMPH *Lymphocytes/leukocytes, *Mono *Monocytes/leukocytes, *Eosino *Eosinophils/leukocytes, *Baso *Basophils/leukocytes

* *p* < 0.05 and ** *p* < 0.01 indicate significant difference compared with week 0 values using to paired t-test

In the placebo group, Ht levels at week 4 were significantly lower than those at week 0 (− 0.93 [− 1.66, − 0.19] %, *p* = 0.02), and MCV at weeks 4, 8, 12, and 16 was significantly lower compared to that at week 0 (− 0.80 [− 1.55, − 0.05], − 1.30 [− 2.13, − 0.47], − 1.20 [− 2.04, − 0.36], − 1.65 [− 2.64, − 0.66] fl., *p* = 0.04, *p* = 0.004, *p* = 0.008, *p* = 0.002, respectively) and lymphocytes/leukocytes at weeks 8 and 12 were significantly lower than those at week 0 (− 2.91 [− 5.68, − 0.14], − 3.02 [− 5.43, − 0.61] %, *p* = 0.04, *p* = 0.02, respectively). RBC and neutrophils/leukocytes counts in the placebo group at week 12 were significantly higher than those at week 0 (12.95 [4.04, 21.86] 10⁴/µL, 3.12 [0.60, 5.93] %, *p* = 0.007, *p* = 0.02, respectively). Hb levels at week 8 and 12 were significantly higher than those at week 0 (0.23 [0.01, 0.44], 0.29 [0.03, 0.54] g/dL, *p* = 0.04, *p* = 0.03, respectively). MCH levels at week 4 were significantly higher than those at week 0 (0.41 [0.15, 0.68] pg, *p* = 0.004), and MCHC levels at weeks 4, 8, and 16 were significantly higher than those at week 0 (0.75 [0.41, 1.08], 0.50 [0.20, 0.80], 0.53 [0.27, 0.79] %, *p* = 0.0001, *p* = 0.002, *p* = 0.0004, respectively).

Tables 5 and 6, and 7 summarize the results for blood biochemical analysis. The following results present the mean differences with 95%CI. In the MCC1848 group, CRE levels at weeks 4 and 16 were significantly lower than those at week 0 (− 0.04 [− 0.07, − 0.01], − 0.04 [− 0.07, − 0.02] mg/dL, *p* = 0.02, *p* = 0.004, respectively), Compared to the levels at week 0, Na levels were significantly lower at week 16 (− 1.24 [− 2.12, − 0.35] mEq/L, *p* = 0.009), Cl levels were significantly lower at weeks 12 and 16 (− 1.35 [− 2.53, − 0.18], − 2.18 [− 3.34, − 1.01] mEq/L, *p* = 0.03, *p* = 0.001, respectively), and Ca levels were significantly lower at week 16 (− 0.13 [− 0.24, − 0.02] mg/dL, *p* = 0.02). FBG and TB levels at week 8 were significantly lower than those at week 0 (− 3.12 [− 5.85, − 0.39] mg/dL, − 0.12 [− 0.23, − 0.02] mg/dL, *p* = 0.03, *p* = 0.02, respectively). HbA1c levels were significantly higher at weeks 8, 12, and 16 (0.04 [0.01, 0.07], 0.13 [0.09, 0.17], 0.12 [0.05, 0.19] %, *p* = 0.01, *p* = 0.00001, *p* = 0.002, respectively), Compared to the levels at week 0, TC levels were significantly higher at week 16 (13.06 [1.69, 24.43] mg/dL, *p* = 0.03), LDL-C levels were significantly higher at weeks 12 and 16 (7.82 [1.22, 14.43], 12.18 [1.46, 22.89] mg/dL, *p* = 0.02, *p* = 0.03, respectively), and HDL-C levels were significantly higher at weeks 12 and 16 ( 4.18 [0.46, 7.89], 4,06 [0.58, 7.54] mg/dL, *p* = 0.03, *p* = 0.03, respectively).

**Table 5:** Blood biochemistry analysis of proteins, pigment, and enzymes

Variables	Reference range	Group	Week 0	Week 4	Week 8	Week 12	Week 16
n(number)		MCC1848	19	18	17	17	17
		Placebo	20	20	20	20	20
TP (g/dl)	6.7–8.3	MCC1848	7.2 ± 0.3	7.1 ± 0.4	7.1 ± 0.3	7.2 ± 0.4	7.1 ± 0.3
		Placebo	7.2 ± 0.3	7.1 ± 0.3	7.1 ± 0.3	7.1 ± 0.3	7.2 ± 0.3
ALB (g/dl)	3.8–5.2	MCC1848	4.3 ± 0.2	4.3 ± 0.2	4.3 ± 0.2	4.3 ± 0.2	4.2 ± 0.2
		Placebo	4.3 ± 0.3	4.3 ± 0.2	4.3 ± 0.2	4.3 ± 0.2	4.2 ± 0.3**
TB (mg/dl)	0.2–1.2	MCC1848	0.9 ± 0.4	0.8 ± 0.4	0.7 ± 0.3*	0.7 ± 0.2	0.7 ± 0.3
		Placebo	0.9 ± 0.3	0.8 ± 0.3	0.8 ± 0.3*	0.8 ± 0.2*	0.8 ± 0.2*
AST (U/L)	10–40	MCC1848	22.5 ± 14.5	19.3 ± 5.4	20.4 ± 7.0	20.8 ± 5.3	21.2 ± 5.2
		Placebo	16.9 ± 3.0	17.9 ± 4.3	18.5 ± 5.4	19.2 ± 6.0*	18.3 ± 4.5
ALT (U/L)	5–45	MCC1848	21.2 ± 26.7	17.2 ± 9.3	17.7 ± 11.2	19.9 ± 14.4	20.2 ± 11.7
		Placebo	12.9 ± 5.1	14.8 ± 5.3*	14.1 ± 6.6	15.2 ± 8.0	16.1 ± 5.8**
LD (U/L)	124–222	MCC1848	174.3 ± 25.8	171.6 ± 23.8	165.5 ± 19.9	173.6 ± 20.9	176.8 ± 30.8
		Placebo	167.2 ± 26.1	166.2 ± 31.3	168.3 ± 48.0	166.4 ± 29.1	168.6 ± 25.9
ALP (U/L)	38–113	MCC1848	67.4 ± 16.4	66.9 ± 17.7	66.2 ± 17.8	66.2 ± 17.7	66.8 ± 16.9
		Placebo	65.4 ± 13.7	65.3 ± 14.4	64.5 ± 13.5	65.3 ± 12.8	68.9 ± 16.1*
γ-GT (U/L)	M: ≤80	MCC1848	20.9 ± 14.0	21.9 ± 13.9	21.2 ± 11.6	23.6 ± 15.0	24.1 ± 13.8
	F: ≤30	Placebo	20.8 ± 12.9	21.4 ± 15.2	19.2 ± 11.6	20.0 ± 11.8	24.3 ± 17.4

Data represent means ± standard deviations

*Abbreviations*: *ALB *Albumin, *ALP *Alkaline phosphatase, *ALT *Alanine aminotransferase, *AST *Aspartate transaminase, *F *Female, *LD *Lactate dehydrogenase, *M *Male, *TB *Total bilirubin, *TP *Total protein, *γ-GT *γ-glutamyl transferase

* *p* < 0.05 and ** *p* < 0.01 indicate significant difference compared with week 0 values using paired t-test

**Table 6:** Blood biochemistry analysis of low molecular nitrogen compounds, carbohydrates, and lipids

Variables	Reference range	Group	Week 0	Week 4	Week 8	Week 12	Week 16
n(number)		MCC1848	19	18	17	17	17
		Placebo	20	20	20	20	20
BUN (mg/dl)	8.0–20.0	MCC1848	13.5 ± 3.8	12.6 ± 3.7	13.5 ± 3.7	13.2 ± 2.9	13.4 ± 4.2
		Placebo	12.3 ± 3.5	11.1 ± 2.7*	12.7 ± 3.4	12.9 ± 3.9	12.6 ± 3.6
CRE (mg/dl)	M: 0.61–1.04	MCC1848	0.75 ± 0.09	0.72 ± 0.11*	0.72 ± 0.12	0.72 ± 0.11	0.71 ± 0.10**
	F: 0.47–0.79	Placebo	0.75 ± 0.15	0.71 ± 0.14**	0.73 ± 0.15	0.72 ± 0.14	0.71 ± 0.15**
UA (mg/dl)	M: 3.8–7.0	MCC1848	4.8 ± 1.0	4.7 ± 0.9	4.8 ± 1.0	4.7 ± 1.1	4.8 ± 1.1
	F: 2.5–7.0	Placebo	4.4 ± 1.1	4.4 ± 1.2	4.5 ± 1.0	4.5 ± 1.1	4.6 ± 1.1
FBG (mg/dl)	70–109	MCC1848	84.2 ± 8.2	83.3 ± 6.6	82.5 ± 9.3*	82.8 ± 10.2	88.4 ± 8.5
		Placebo	85.8 ± 6.1	85.6 ± 4.7	83.8 ± 5.4	85.0 ± 5.1	86.8 ± 6.8
HbA1c (%)	4.6–6.2	MCC1848	5.3 ± 0.3	5.3 ± 0.3	5.4 ± 0.3*	5.5 ± 0.3**	5.4 ± 0.3**
		Placebo	5.4 ± 0.2	5.4 ± 0.2	5.4 ± 0.2	5.4 ± 0.2*	5.4 ± 0.2**
TC (mg/dl)	120–219	MCC1848	212.1 ± 37.4	215.3 ± 38.8	212.1 ± 39.2	218.8 ± 35.2	225.3 ± 47.1*
		Placebo	205.5 ± 35.9	206.3 ± 37.2	207.0 ± 32.8	210.6 ± 34.4	213.9 ± 37.8*
LDL-C (mg/dl)	65–139	MCC1848	121.8 ± 30.5	129.5 ± 33.5	126.9 ± 32.7	129.8 ± 32.5*	134.2 ± 42.6*
		Placebo	122.1 ± 33.5	125.0 ± 37.5	125.5 ± 32.1	128.2 ± 31.5	131.1 ± 36.6*
HDL-C (mg/dl)	M: 40–85	MCC1848	70.2 ± 16.9	70.6 ± 16.5	70.1 ± 16.8	73.4 ± 16.3*	73.2 ± 19.4*
	F: 40–95	Placebo	64.6 ± 11.2	66.0 ± 12.5	65.9 ± 12.9	68.7 ± 15.7*	63.5 ± 14.0
TG (mg/dl)	30–149	MCC1848	82.2 ± 47.7	80.8 ± 36.3	82.8 ± 41.2	89.9 ± 47.9	76.2 ± 31.7
		Placebo	75.6 ± 50.4	74.3 ± 35.6	80.8 ± 48.0	81.7 ± 48.7	80.1 ± 55.9

Data represent means ± standard deviations

*Abbreviations*: *BUN *Blood urea nitrogen, *CRE *Creatinine, *F *Female, *FBG *Fasting blood glucose, *HbA1c *Hemoglobin A1c, *HDL-C *High-density lipoprotein cholesterol, *LDL-C *Low‐density lipoprotein cholesterol, *M *Male, *TC *Total cholesterol, *TG *Triglyceride, *UA *Uric acid. * *p* < 0.05 and ** *p* < 0.01 indicate significant difference compared with week 0 values using to paired t-test

**Table 7:** Blood biochemistry analysis of electrolytes and trace element

Variables	Reference range	Group	Week 0	Week 4	Week 8	Week 12	Week 16
n(number)		MCC1848	19	18	17	17	17
		Placebo	20	20	20	20	20
Na (mEq/L)	137–147	MCC1848	141.6 ± 1.6	141.6 ± 1.8	141.4 ± 1.5	141.4 ± 1.5	140.6 ± 1.7**
		Placebo	140.9 ± 1.7	141.5 ± 1.8	140.6 ± 1.8	140.8 ± 1.6	139.5 ± 1.7**
Cl (mEq/L)	98–108	MCC1848	105.6 ± 2.3	105.2 ± 1.8	105.3 ± 1.2	104.6 ± 1.4*	103.8 ± 1.8**
		Placebo	104.8 ± 2.0	105.4 ± 2.2	104.8 ± 2.5	103.7 ± 1.7*	102.9 ± 1.8**
K (mEq/L)	3.5–5.0	MCC1848	4.3 ± 0.3	4.3 ± 0.2	4.3 ± 0.3	4.3 ± 0.3	4.1 ± 0.3
		Placebo	4.1 ± 0.3	4.1 ± 0.3	4.2 ± 0.3	4.2 ± 0.4	4.1 ± 0.3
Ca (mg/dl)	8.4–10.4	MCC1848	9.4 ± 0.3	9.3 ± 0.3	9.2 ± 0.3	9.4 ± 0.2	9.3 ± 0.2*
		Placebo	9.3 ± 0.3	9.3 ± 0.2	9.2 ± 0.2*	9.3 ± 0.3	9.2 ± 0.3

Data represent means ± standard deviations

*Na *sodium, Cl Chloride, *K *Potassium, *Ca *Calcium

* *p* < 0.05 and ** *p* < 0.01 indicate significant difference compared with week 0 values using to paired t-test

In the placebo group, compared to the levels at week 0, ALB and Na levels were significantly lower at week 16 (− 0.16 [− 0.24, − 0.07] g/dL, − 1.45 [− 2.14, − 0.76] mEq/L, *p* = 0.001, *p* = 0.0003, respectively), TB levels were significantly lower at weeks 8, 12, and 16 (− 0.12 [− 0.21, − 0.02], − 0.11 [− 0.220, − 0.001], − 0.12 [− 0.22, − 0.01] mg/dL, *p* = 0.022, *p* = 0.049, *p* = 0.039, respectively), BUN levels were significantly lower at week 4 (− 1.18 [− 2.29, − 0.07] mg/dL, *p* = 0.038), CRE levels were significantly lower at weeks 4 and 16 (− 0.04 [− 0.06, − 0.02], − 0.04 [− 0.06, − 0.02] mg/dL, *p* = 0.001, *p* = 0.001, respectively), Cl levels were significantly lower at weeks 12 and 16 (− 1.10 [− 1.97, − 0.23], − 1.95 [− 2.80, − 1.10] mEq/L, *p* = 0.016, *p* = 0.0001, respectively), and Ca levels were significantly lower at week 8 (− 0.08 [− 0.16, − 0.01] mg/dL, *p* = 0.037). Furthermore, in the placebo group, compared to the levels at week 0, AST and HDL-C levels were significantly higher at week 12 (2.35 [0.12, 4.58] U/L, 4.10 [0.30, 7.90] mg/dL, *p* = 0.04, *p* = 0.04, respectively), ALT levels were significantly higher at weeks 4 and 16 (1.90 [0.24, 3.56], 3.20[1.66, 4.74] U/L, *p* = 0.03, *p* = 0.0003, respectively), ALP, TC, and LDL-C levels were significantly higher at week 16 (3.50 [0.27, 6.73] U/L, 8.40 [0.47, 16.33] mg/dL, 8.95 [0.97, 16.93] mg/dL, *p* = 0.04, *p* = 0.04, *p* = 0.03, respectively), and HbA1c levels were significantly higher at weeks 12 and 16 ( 0.04 [0.002, 0.078], 0.06 [0.025, 0.095] %, *p* = 0.042, *p* = 0.002, respectively).

Table 8 summarizes the measurement results of USG and U-pH. There were no significant differences in USG and U-pH between or within the groups. Similarly, there were no significant differences in U-pro, U-glu, U-uro, U-bil, U-ket, and OBR between the groups (Table 9).

**Table 8:** Summary of urine specific gravity and urine pH

Variables	Reference range	Group	Week 0	Week 4	Week 8	Week 12	Week 16
n(number)		MCC1848	19	18	17	17	17
		Placebo	20	20	20	20	20
USG	1.006–1.030	MCC1848	1.015 ± 0.006	1.014 ± 0.007	1.017 ± 0.006	1.017 ± 0.006	1.018 ± 0.007
		Placebo	1.015 ± 0.008	1.016 ± 0.008	1.019 ± 0.007	1.017 ± 0.007	1.019 ± 0.009
U-pH	5.0–7.5	MCC1848	6.0 ± 0.5	6.1 ± 0.6	6.1 ± 0.5	6.3 ± 0.9	6.0 ± 0.5
		Placebo	6.2 ± 0.5	6.1 ± 0.7	6.3 ± 0.4	6.1 ± 0.6	6.0 ± 0.6

Data indicate means ± standard deviations

*USG *Urine specific gravity, *U-pH *Urine pH

**Table 9:** Summary of urinalysis results

Variables	Week	MCC1848week 0 : *n* = 19week 4 : n = 18week 8, 12, 16 :n = 17		Placebo *n* = 20		*p* value
		Reference range		Reference range		
		Within	Outside	Within	Outside	
U‐pro	0	18	1	19	1	1
	4	17	1	20	0	0.474
	8	17	0	19	1	1
	12	17	0	20	0	NA
	16	16	1	18	1	1
U‐glu	0	19	0	19	1	1
	4	18	0	19	1	1
	8	17	0	19	1	1
	12	16	1	20	0	0.459
	16	17	0	20	0	NA
U‐uro	0	18	1	20	0	0.487
	4	18	0	20	0	NA
	8	17	0	20	0	NA
	12	17	0	20	0	NA
	16	16	1	20	0	0.459
U‐bil	0	19	0	20	0	NA
	4	18	0	20	0	NA
	8	17	0	20	0	NA
	12	17	0	20	0	NA
	16	17	0	20	0	NA
U‐ket	0	19	0	20	0	NA
	4	18	0	20	0	NA
	8	17	0	20	0	NA
	12	17	0	20	0	NA
	16	17	0	20	0	NA
OBR	0	18	1	19	1	1
	4	18	0	20	0	NA
	8	16	1	19	1	1
	12	17	0	20	0	NA
	16	16	1	19	1	1

Data represent the number of subjects

*Abbreviations*: *NA *Not applicable, *OBR *Occult blood reaction, *U-bil *Urine biliru-bin, *U-glu *Urine glucose, *U-ket *Urine ketone body

*p* values were calculated using Fisher's exact test; U-pro, urine protein; U-uro, urine urobilinogen

### AEs and side effects

Throughout the study, 10 participants reported a total of 11 AEs. Six AEs were reported by six participants in the MCC1848 group, and five AEs were reported by four participants in the placebo group. These AEs included scalp rash (one participant in the MCC1848 group), common cold (three participants in the placebo group), toe contusion (one participant in the MCC1848 group), influenza (two participants in the MCC1848 group), left elbow fracture (one participant in the placebo group), arrhythmia (one participant in the MCC1848 group), toothache (one participant in the MCC1848 group) and acute upper respiratory tract infection (one participant in the placebo group). There was no significant difference in the incidence of AEs between the MCC1848 and placebo intake groups (*p* = 0.480). All the reported AEs were mild and determined to be “Not related” to the intake of study products (Intervention end date: December 14, 2024, Follow-up end date: January 18, 2025). Importantly, no gastrointestinal adverse events such as diarrhea, abdominal pain, or bloating were reported in either group during the study period.

## Discussion

In this study, we meticulously evaluated the safety profile of consuming 25 billion *L. helveticus* MCC1848 daily over a 12-week period in healthy adults. Our comprehensive safety assessment encompassed blood pressure measurements, hematological evaluations, biochemical analyses, urine tests, and detailed medical interviews. All the AEs were classified as incidental and unrelated to the test substance by the Principal Investigator. Throughout the study duration, 11 AEs were documented from 10 participants. Specifically, within the *L. helveticus* MCC1848 group, 6 AEs were reported from 6 participants, all of which were mild, transient, and deemed unrelated to the study intervention. These findings suggest a robust safety profile for the high-dose consumption of *L. helveticus* MCC1848. In particular, the absence of gastrointestinal complaints or abnormalities in urinalysis further supports the favorable tolerability of heat‑killed *L. helveticus* MCC1848 when consumed at this high dose over 12 weeks. Although the MCC1848 group exhibited a significantly higher DBP at baseline compared with the placebo group, this difference diminished during the intervention, and no between‑group differences were detected at the final assessment. Given that analyses focused on both within‑group changes over time and between‑group comparisons, this baseline imbalance is unlikely to have materially affected the interpretation of the safety outcomes. Several hematological and biochemical parameters showed statistically significant changes during the intervention period in one or both groups; however, all measured values remained within their respective physiological reference ranges throughout the study. The principal investigator carefully reviewed all laboratory findings while maintaining blinding and judged that none of these fluctuations were clinically meaningful or indicative of any safety concerns. Accordingly, these statistically significant variations are considered to have no adverse impact on the overall safety assessment.

*L. helveticus*, the species to which MCC1848 belongs, has been extensively used in various food products, including dairy items, fermented and aged meats, and fermented vegetables [12]. Owing to its enduring safe usage, *L. helveticus* is classified as safe under the Qualified Presumption of Safety (QPS) framework established by the European Food Safety Authority [10]. Although the QPS list typically pertains to live microorganisms, the food used in this study was heat-killed and non-proliferative, thereby minimizing the risk of bacteremia [13]. The *L. helveticus* MCC1848 used in this study was heat‑killed and therefore does not exhibit any metabolic activity, making it unlikely to pose an increased safety risk. Whereas previous randomized controlled trials using heat‑killed *L. helveticus* MCC1848 primarily evaluated mood‑related outcomes at a daily dose of 5 billion cells for 4 weeks [8], the present study expands upon this evidence base by assessing a five‑fold higher dose administered over a substantially longer duration. The absence of clinically meaningful alterations in anthropometric measures, blood pressure, hematological and biochemical parameters, and urinalysis—combined with the low and comparable incidence of adverse events between groups—demonstrates that escalating the dose to 25 billion cells per day for 12 weeks does not introduce additional safety concerns in healthy adults. Collectively, these findings support the feasibility of utilizing heat‑killed *L. helveticus* MCC1848 as a postbiotic ingredient aligned with GRAS‑ and QPS‑based safety expectations for applications in food products.

Despite the robust design of this study to evaluate the safety of high-dose, long-term consumption of *L. helveticus* MCC1848, certain limitations should be acknowledged. This study was conducted exclusively in healthy adults, which limits the generalizability of the safety findings. Because individuals with underlying health conditions were not included, our results cannot be directly extrapolated to populations with comorbidities or chronic diseases, who may have different safety profiles. Further studies in such populations will be necessary to confirm the safety of long-term intake of heat-killed *L. helveticus* MCC1848 under broader clinical conditions. Moreover, detailed quantitative monitoring of physical activity was not performed. Participants were instructed to maintain their usual habits, and diaries and interviews were used to confirm the absence of major lifestyle changes; however, residual confounding by unmeasured behaviors cannot be fully excluded.

## Conclusions

This randomized, double-blind, placebo-controlled trial provides compelling evidence for the safety of consuming 25 billion heat-killed *L. helveticus* MCC1848 daily for 12 weeks in healthy adults. No AEs related to the intake were observed in anthropometric and blood pressure measurements, blood and urine analyses, or medical interviews. Thus, the long-term intake of 25 billion heat-killed *L. helveticus* MCC1848 is safe for healthy adults.

## Data Availability

The data used and analyzed during the current study are available from the corresponding author upon reasonable request.

## Abbreviations

AE: Adverse event
ALB: Albumin
ALP: Alkaline phosphatase
ALT: Alanine aminotransferase
AST: Aspartate transaminase
BMI: Body mass index
BUN: Blood urea nitrogen
BW: Body weight
CRE: Creatinine
DBP: Diastolic blood pressure
FBG: Fasting blood glucose
Hb: Hemoglobin
HbA1c: Hemoglobin A1c
HDL-C: HDL-cholesterol
IFCC: Lactate dehydrogenase
LDL-C: LDL-cholesterol
MCH: Mean corpuscular hemoglobin
MCHC: Mean corpuscular hemoglobin concentration
MCV: Mean corpuscular volume
OBR: Occult blood reaction
QOL: Quality of life
QPS: Qualified Presumption of Safety
RBC: Red blood cell count
SAF: Safety analysis set
SBP: Systolic blood pressure
TB: Total bilirubin
TC: Total cholesterol
U-bil: Urine bilirubin
U-glu: Urine glucose
U-ket: Urine ketones
U-pH: Urine ph
U-pro: Urine protein
USG: Urine specific gravity
U-uro: Urine urobilinogen
